# Absolute binding-free energies between standard RNA/DNA nucleobases and amino-acid sidechain analogs in different environments

**DOI:** 10.1093/nar/gku1344

**Published:** 2014-12-30

**Authors:** Anita de Ruiter, Bojan Zagrovic

**Affiliations:** Department of Structural and Computational Biology, Max F. Perutz Laboratories, University of Vienna, Vienna 1030, Austria

## Abstract

Despite the great importance of nucleic acid–protein interactions in the cell, our understanding of their physico-chemical basis remains incomplete. In order to address this challenge, we have for the first time determined potentials of mean force and the associated absolute binding free energies between all standard RNA/DNA nucleobases and amino-acid sidechain analogs in high- and low-dielectric environments using molecular dynamics simulations and umbrella sampling. A comparison against a limited set of available experimental values for analogous systems attests to the quality of the computational approach and the force field used. Overall, our analysis provides a microscopic picture behind nucleobase/sidechain interaction preferences and creates a unified framework for understanding and sculpting nucleic acid–protein interactions in different contexts. Here, we use this framework to demonstrate a strong relationship between nucleobase density profiles of mRNAs and nucleobase affinity profiles of their cognate proteins and critically analyze a recent hypothesis that the two may be capable of direct, complementary interactions.

## INTRODUCTION

From processing, transport and translation of mRNA to transcription and modification of DNA, many different processes in the cell critically depend on direct, specific interactions between proteins and nucleic acids (1). Despite the clear biological importance of such interactions, however, our understanding of the basic physico-chemical principles that define them at the atomistic level remains incomplete. This in particular concerns the very foundation of nucleic acid–protein interactions, that is, the intrinsic binding preferences of nucleobases and amino acids for each other in different environments. When it comes to experimental work, for example, only limited progress has been made in this context. Akinrimisi *et al.* (2) and Thomas *et al.* (3) have used spectroscopic methods to study the change in water solubility of a subset of amino acids in the presence of either purine molecules or different nucleosides, respectively. Moreover, Thomas *et al.* have in this way also determined association constants for several amino acid and nucleoside pairs (3). In addition, Woese *et* *al*. have used chromatographic methods to systematically study interaction propensities of all 20 common amino acids and different pyridine derivatives in water (4–6).

In addition, significant information on the type of interactions, their strengths and preferred geometries at the single-molecule, atomic-resolution level has been obtained through computational analysis. The majority of previous studies in this context belong to two main classes. First, analysis of high-resolution 3D structures of protein–RNA or protein–DNA complexes has produced valuable information on the relative binding preferences of amino-acid sidechains and nucleobases for each other together with a geometric and energetic characterization of their interactions (7–16). Second, quantum-mechanical *ab initio* calculations have been applied to study the physical aspects of such binding, including nucleobase-amino acid π–π (17–21) and cation–π interactions (22,23) and hydrogen bonding (24). Moreover, there have also been reports on the free energy maps for the binding of a smaller subset of amino acids to DNA base pairs (25,26). In general, however, the great majority of both experimental and computational studies have focused on specific binding interactions and typically covered a small subset of amino acid and nucleobase types only. In particular, despite their fundamental importance, absolute free energies of binding between all standard nucleobases and amino acids have never before been determined in a systematic, self-consistent manner.

A particular context in which direct interactions between nucleobases and amino acids may be important concerns the origin of the universal genetic code, one of the most important foundational questions in molecular biology that are still open (5,27–35). More specifically, the stereochemical hypothesis (5,31,34,35) postulates that the code evolved as a consequence of direct binding preferences of amino acids for their cognate codons. Although highly suggestive and biologically reasonable, evidence supporting the hypothesis in its one-amino-acid/one-codon formulation has nonetheless been obtained for a small subsection of the genetic code only (31,34,35). Recently, we have presented evidence in support of a generalization of the stereochemical hypothesis and suggested that under some circumstances cognate mRNA and protein sequences may, in fact, be mutually physico-chemically complementary to each other and bind (36–38). In this framework, binding specificity and an appreciable level of interaction appear primarily at the level of longer mRNA and protein stretches. In particular, we have shown that pyrimidine-density profiles of typical, present-day mRNA sequences exhibit strong correlation with cognate proteins’ sequence profiles capturing their interaction propensity with pyrimidine mimetics (36). Moreover, we have derived interaction preference scales for nucleobases and amino-acid sidechains by analyzing binding interfaces in a large set of 3D structures of RNA–protein complexes (37). By comparing the nucleobase-content profiles of mRNA sequences with the nucleobase-preference-weighted profiles of their cognate protein sequences, we have found strong evidence for the complementarity hypothesis, but also demonstrated exceptions from it in some cases. For example, we found that purine density in mRNA sequences correlates directly with guanine preference profiles of their cognate protein sequences, yet inversely with the equivalent adenine preference profiles.

Here, we use Molecular Dynamics (MD) simulations and umbrella sampling (US), in conjunction with a detailed comparison against extant experimental data, to determine for the first time potentials of mean force (PMFs) and absolute binding free energies between all possible combinations of standard RNA/DNA nucleobases and non-prolyl/non-glycine amino-acid sidechain analogs. Sidechain analogs have been widely used instead of complete amino acids for testing the interaction specificity of amino-acid residues in different contexts (20,26,39). There are several advantages to such a choice. First, zwitterionic amino acids contain charged groups which, apart from the N- and C-terminal residues, are not present in proteins. Second, capping of amino acids necessarily introduces groups that do not represent the actual protein backbone. Finally, sidechain analogs have been used to parameterize GROMOS 54a8, arguably the most accurate classical force field when it comes to capturing amino-acid hydrophobicity (40,41). As nucleobase/amino acid interactions are strongly influenced by the hydrophobic effect, a combination of the GROMOS 54a8 force field and sidechain analogs suggested itself as a particularly suitable choice. On the other hand, a clear disadvantage of using sidechain analogs is that glycine and proline cannot be treated in the same way as other amino acids. Moreover, it has been shown that the backbone contribution to amino-acid solvation free energies does vary between different amino acids because of self-solvation effects (42). However, as self-solvation arises primarily in the gas phase (42), this becomes less relevant in our context.

In order to study the effect of the environment on the binding preferences, our US simulations are performed in both water and methanol. The latter is chosen based on its lower dielectric constant, which is expected to capture the environment at nucleic acid-protein interfaces more accurately than pure water (43,44). Overall, our analysis opens up a microscopically detailed perspective on nucleobase/sidechain interactions, and provides a self-consistent platform for understanding and designing nucleic acid-protein interactions in different settings. Here, we apply the newly obtained binding preferences to study the putative physico-chemical foundation of the universal genetic code and critically examine the cognate mRNA–protein complementarity hypothesis (36–38).

## MATERIALS AND METHODS

### MD simulations

All MD simulations were conducted using the GROMOS11 simulation package (45) in combination with the GROMOS force field parameter set 54a8 (41). Bond lengths were constrained by applying the SHAKE algorithm (46) with a relative geometric accuracy of 10^−4^, allowing for a time step of 2 fs. RNA nucleobases were methylated at N1 (pyrimidines) and N9 (purines) positions, whereas amino-acid sidechains were capped by a hydrogen atom at C_β_, in order to focus on specific interactions and to aid sampling. In this manner, 18 out of the 20 natural occurring amino acids could be studied (all except glycine and proline). The amino acids arginine, aspartate, glutamate, lysine and histidine were represented in their charged forms at pH 7. In addition, histidine was simulated in both neutral states (HISA, HISB) as well. Each nucleobase/sidechain pair was placed at a large distance from each other and subsequently solvated in a periodic cubic box with Simple Point Charge (SPC) (47) water molecules. Further details concerning MD are given in Supplementary Information.

### Umbrella sampling

The reaction coordinate *r* for the potential of mean force was defined as the distance between the center of geometry (cog) of the nucleobase and the cog of the amino-acid sidechain analog. Sampling along this reaction coordinate was enhanced by performing US, where harmonic distance restraints with a force constant of 500 kJ mol^−1^ nm^−2^ were used as biasing potentials. The restraining simulations were performed sequentially, i.e. after a short equilibration time of 100 ps, the production run of 10 ns at this distance was started, followed by the equilibration at a smaller separation and so on. In this way, the molecules were slowly pulled closer together. Further details concerning the calculation of PMFs and free energy differences using US, comparison of mRNA and protein sequences and the estimation of statistical significance are given in the Supplementary Information.

## RESULTS

### PMFs and binding free energies

**Figure 1.**
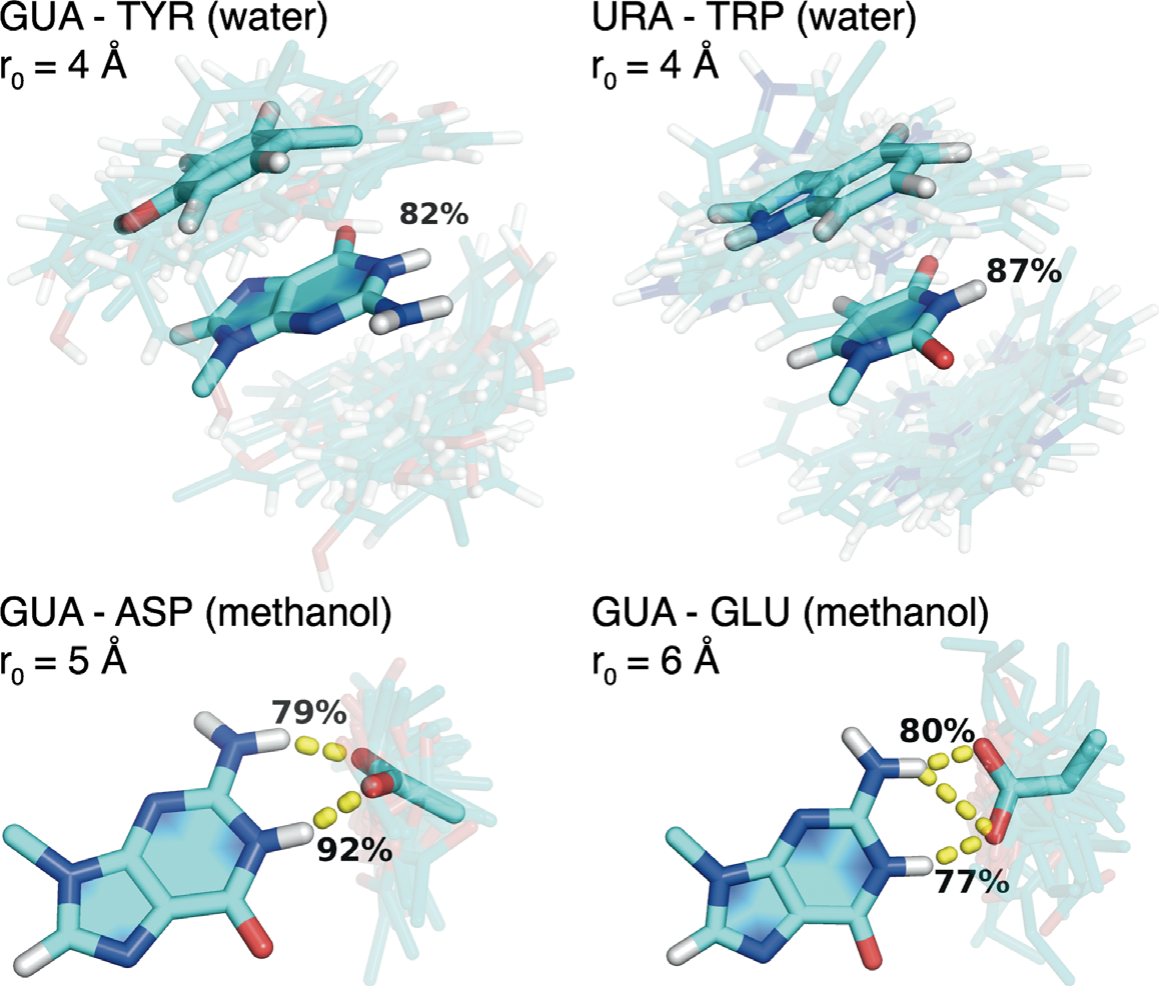
Sampling of binding modes for the pairs GUA–TYR (4 Å) and URA–TRP (4 Å) in water as well as GUA–ASP (5 Å) and GUA–GLU (6 Å) in methanol at a restraining distance *r*_0_ corresponding to a PMF minimum. Centers of the most populated cluster are shown in an opaque stick representation, while snapshots of sidechain analogs output every 200 ps are shown in a transparent stick representation. Percentages represent fraction of time that stacking interactions (TYR or TRP) or hydrogen bonds (ASP and GLU) are present.

At each restraining distance used in US simulations, nucleobases and sidechain analogs explore different configurations, with shorter distances expectedly resulting in more restricted configurational diversity. In Figure 1, we illustrate configurational ensembles of four nucleobase/sidechain pairs for which the resulting binding free energies are the lowest over all simulated conditions, as discussed below: GUA–TYR and URA–TRP in water and GUA–ASP and GUA–GLU in methanol. In particular, for each such nucleobase and sidechain analog pair, we present the configurational diversity at the restraining distance *r*_0_ corresponding to the minimum in the respective PMF curve (see below). In addition, we have clustered all of the structures sampled at this distance using root-mean-square deviation as the metric and determined the center of the largest cluster. These structures are shown in opaque for each nucleobase/sidechain pair in Figure 1 as a representation of the predominant geometric orientation. Overall, there are two principal contributions stabilizing the most favorable complexes as illustrated for select cases in Figure 1: in water simulations, these are typically stacking interactions between aromatic groups (as illustrated in Figure 1 for GUA–TYR and URA–TRP pairs), while in methanol simulations, these are strong H-bonding interactions (as illustrated in Figure 1 for GUA–ASP and GUA–GLU).

**Figure 2.**
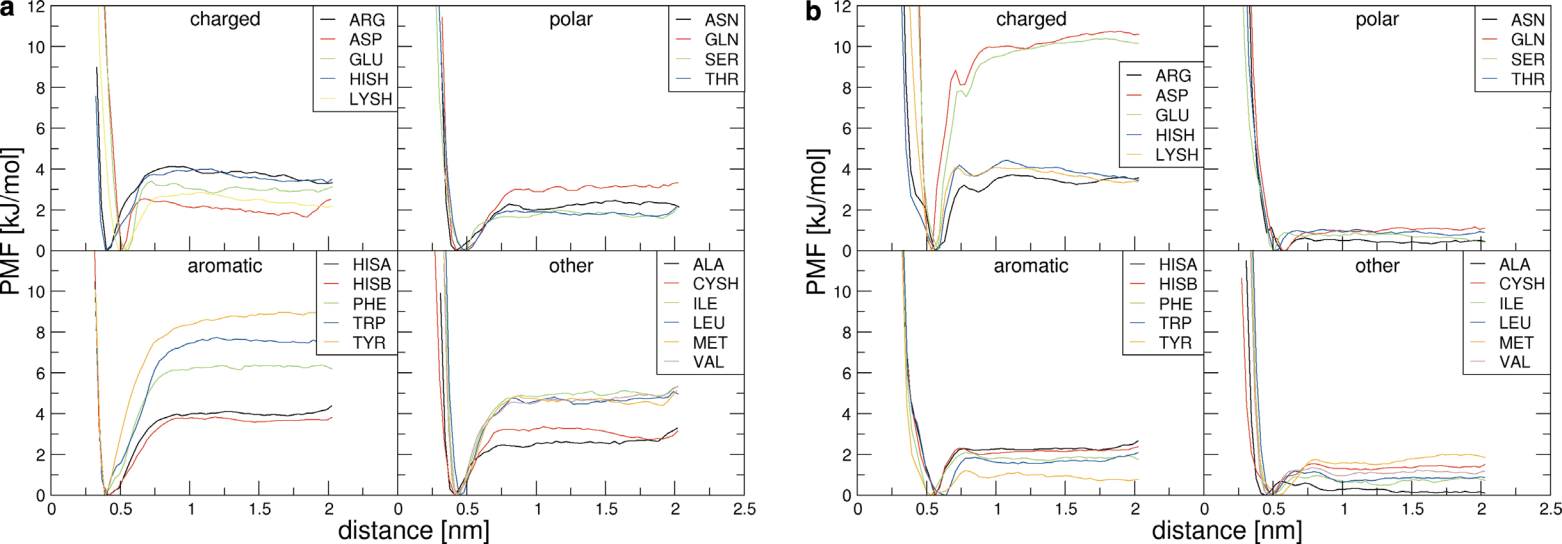
Potentials of mean force for GUA with all sidechain analogs in water (**a**) and in methanol (**b**).

A quantitative way of capturing the behavior of individual pairs of nucleobases and sidechain analogs is by analyzing PMFs. In particular, PMFs give an overview of binding preferences corresponding to a given base or sidechain, demonstrate differences in the optimal binding distance and help identify energetic barriers along the binding reaction coordinate. Given its peculiar behavior, the PMF curves for GUA with each of the sidechain analogs simulated in water and methanol are shown in Figure 2a and b, respectively, with all other PMFs given in Supplementary Figure S1. Overall, one can see that in water, all nucleobases exhibit a pronounced preference for the aromatic TYR and TRP, followed closely by PHE. Weaker interactions with nucleobases are observed for other hydrophobic sidechains, while the weakest binding is exhibited by charged and polar sidechains (Figure 2a and Supplementary Figure S1). Altogether, there appears to be significant qualitative similarity in the way different nucleobases and sidechain analogs interact in water: in addition to their preference for aromatic sidechains, none of the PMF curves in water show any significant energy barriers regardless of the base in question (Figure 2a and Supplementary Figure S1).

On the other hand, the influence of the environment on the binding preferences of sidechains and nucleobases, and in particular GUA, becomes immediately clear if one compares the PMFs in water (Figure 2a and Supplementary Figure S1) with those in methanol (Figure 2b and Supplementary Figure S1). There, contrary to its behavior in water, GUA exhibits a strong preference for the negatively charged GLU and ASP over aromatic sidechains (Figure 2b). On the other hand, all other nucleobases in methanol show weak, if any, preference for different sidechains (Supplementary Figure S1). In addition, the minima of the PMF curves typically shift to larger distances in methanol (Figure 2 and Supplementary Figure S1). Although this can be observed for most sidechains, the largest shifts are seen for aromatics. Since the distances in the PMFs are defined between centroids of nucleobases and sidechains, the smallest distance between a given nucleobase and an aromatic sidechain can be obtained in π–π stacked geometry. The fact that intermolecular distances in methanol are typically larger than in water suggests that stacking interactions are destabilized in the former, especially for ADE, GUA and CYT. Analysis of residence times in stacked configurations corroborates this qualitative observation (Table 1). For example, while at the optimal distance for binding in water, TRP and TYR spend 96 and 94% of time in stacked configurations with GUA, these numbers drop to 7 and 18% of time in methanol. Similarly, PHE spends 85% of the time in stacked configuration with CYT in water, a number which drops to 13% in methanol. This phenomenon has already been observed in experiment and other simulations and can be explained by the inhibitory effect of the methyl group of the solvent on the solute–solute dispersive interactions (48,49). On the other hand, these differences are much less drastic for URA and THY (Table 1). Finally, apart from the differences in the apparent binding preferences, the PMF curves also differ when it comes to well-defined energetic barriers, which although small, can be found in methanol simulations, but not in water simulations (Figure 2 and Supplementary Figure S1).

**Table 1.** Stacking interactions in water and methanol simulations

	ADE				CYT				GUA				URA				THY			
	wat		met		wat		met		wat		met		wat		met		wat		met	
	*R*_0_	%	*R*_0_	%	*R*_0_	%	*R*_0_	%	*R*_0_	%	*R*_0_	%	*R*_0_	%	*R*_0_	%	*R*_0_	%	*R*_0_	%
HISA	4	56	5	13	3	79	5	6	4	62	5	11	3	84	5	12	3	87	3	79
HISB	4	45	5	9	3	71	5	7	3	73	5	9	3	80	5	10	3	84	6	1
HISH	3	88	3	83	4	52	5	12	3	84	6	4	4	1	6	1	4	1	6	0
PHE	5	24	5	10	3	85	5	13	3	88	5	13	3	91	3	83	2	98	3	89
TRP	5	35	6	5	3	91	5	21	3	96	6	7	4	88	3	93	2	99	2	99
TYR	3	91	5	16	3	90	5	14	3	94	5	18	3	93	3	87	4	85	3	90

Percentage of time that stacking interactions are present during simulation as shown for the window around the minimum of the PMF, as determined by the restraining distance R_0_.

While PMF curves are undeniably informative, the most complete information concerning binding preferences of bases and sidechain analogs and their dependence on the environment can be obtained by examining absolute binding free energies derived from PMFs (Table 2). Overall, the free energies in water are relatively low across the board, with the most favorable values seen in the case of THY–TRP (-6.1 kJ/mol, Table 2a). The strongest interactions for all the nucleobases in water are in general those with aromatic sidechains, with PHE exhibiting somewhat less favorable binding free energies compared to TRP and TYR (Table 2a). Similarly, for all the nucleobases, interactions with non-aromatic hydrophobic sidechains appear to be slightly favorable in water (ca. −3 to −1 kJ/mol). On the other hand, the only significantly unfavorable interactions in water are those between the nucleobases and the negatively charged GLU and ASP, with the only outlier in this sense being a slightly favorable interaction between GUA and GLU (−0.3 kJ/mol) (Table 2a). On the whole, different nucleobases exhibit very similar preferences when it comes to interaction with sidechains in water as best illustrated by Pearson correlation coefficients *R* between different combinations of nucleobase preference scales (columns in Table 2A), which all give values close to or in excess of 0.9 (Supplementary Table S1a).

**Table 2.**
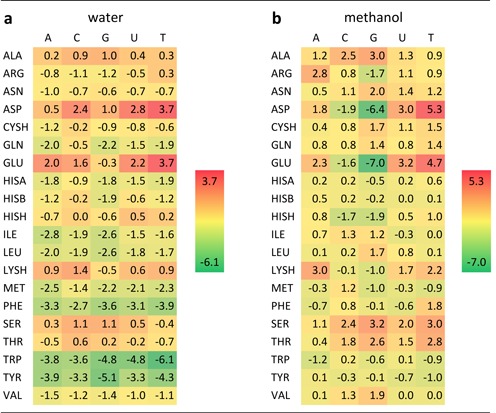
Absolute binding free energies between nucleobases and amino-acid sidechains in water (**a**) and methanol (**b**) in kJ/mol

**Figure 3.**
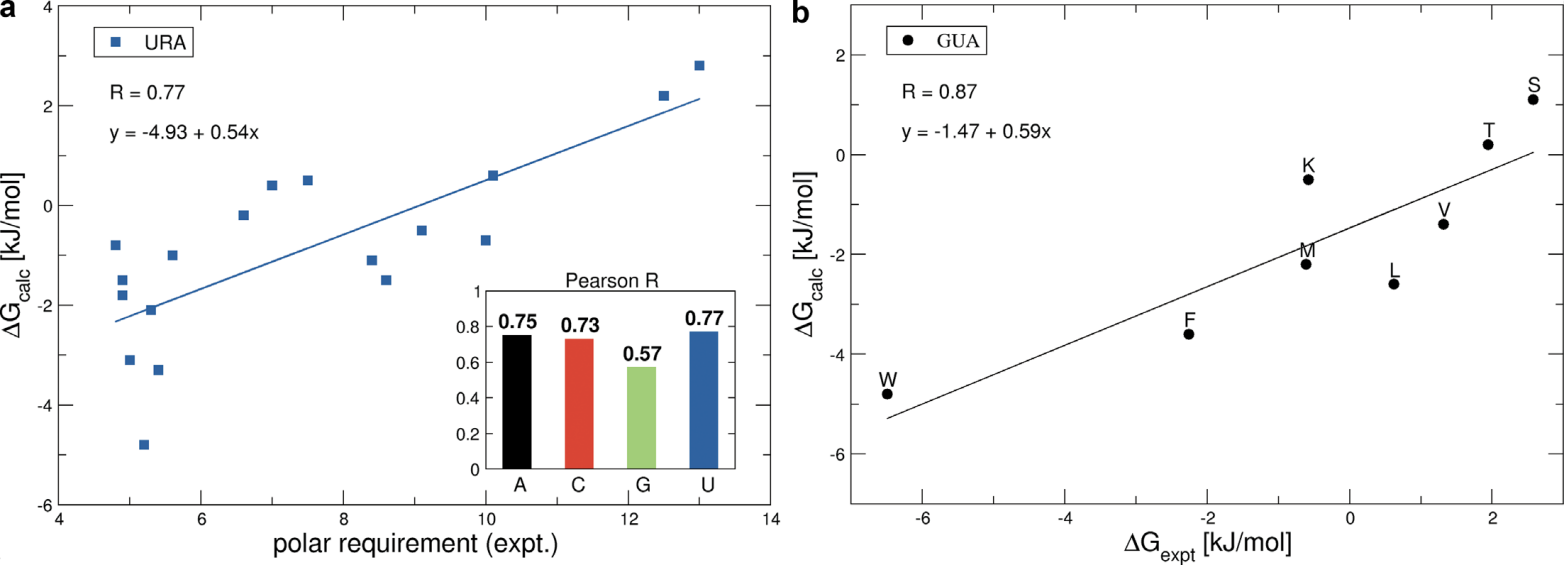
(**a**) Comparison of the computationally derived binding free energies for URA in water with the experimental polar requirement (PR) scale. Inset: pearson *R* correlations between polar requirement and the binding free energy scales in water. (**b**) Correlation between the experimentally determined binding free energies of guanosine with 8 amino acids (3) and the computationally derived binding free energies of guanine with the corresponding 8 amino-acid sidechains in water.

Importantly, all scales in water exhibit pronounced correlation with Woese's experimental PR scale capturing the interaction propensity of amino acids and nucleobase-like 2,6-dimethylpyridine (4–6) as shown in the inset of Figure 3a. For example, the URA scale in water, which is particularly interesting given that uracil is sterically and physico-chemically the most similar natural nucleobase to 2,6-dimethylpyridine, exhibits a Pearson *R* of 0.77 with the experimental PR scale (Figure 3a). What is more, the GUA scale in water exhibits strong agreement with the only extensive experimental scale available involving natural bases, that of association constants between eight amino acids (SER, THR, VAL, LEU, MET, LYS, PHE and TRP) and guanosine as shown in Figure 3b. Here, after the reported association constants (3) have been converted to binding free energies, the Pearson *R* equals 0.87. Moreover, the results do not significantly change if one also includes in this set all the experimentally available values (3) for adenosine (LYS, PHE, TRP, VAL), cytosine (PHE, TRP) and uridine (TRP), with the Pearson *R* against the equivalent computed values of 0.75. Taking into account that substituted pyridines and nucleosides are chemically still quite different from nucleobases themselves, this level of agreement is remarkable and it attests to the quality of our simulation methodology and gives confidence as to its general applicability. More specifically, these results support the possibility that the absolute binding free energies and particularly their relative ranking may be well captured for all other combinations of nucleobases and amino-acid sidechains as well.

How does the above picture change if one examines the binding free energies in methanol? In accordance with the PMF analysis, the only significantly favorable interactions in methanol are seen in the case of GUA and the negatively charged GLU (−7.0 kJ/mol) and ASP (−6.4 kJ/mol), with no other individual interactions being stronger than −2 kJ/mol (Table 2b). In particular, unlike in water, there are no significant interactions with aromatic sidechains for any of the nucleobases in methanol (Table 2). On the other hand, relatively pronounced unfavorable interactions are seen only in the case of THY and ASP and GLU, but even in those cases the binding free energies do not exceed 5.5 kJ/mol. The impact of the environment on the interactions can best be quantified by calculating Pearson *R*s between binding free energy scales in water and in methanol. For the two GUA scales, for example, *R* is close to zero (−0.1), indicating no correlation between the binding free energies in the two environments. A similar situation is seen for CYT (*R* = −0.2), whereas for ADE, URA and THY strong correlations can be found with *R*-values around 0.8 (Supplementary Table S1b). In other words, binding preferences of GUA and CYT change greatly upon the change from an aqueous solvent to a more interface-like, lower dielectric environment, whereas binding preferences of ADE, URA and THY remain largely indifferent to such a change. Finally, strong correlations can be seen between URA and THY (*R* = 0.87), CYT and GUA (*R* = 0.85) and ADE and URA (*R* = 0.67) scales in methanol (Supplementary Table S1c). This is particularly interesting considering the relatively low and noisy values of binding free energies in most scales.

The pronounced dependence of binding preferences of GUA and CYT on the properties of the environment originates predominantly from the contribution of negatively charged sidechains, and to a smaller extent, from aromatic ones (Table 2). The negatively charged ASP and GLU are the strongest binders for both CYT and GUA in methanol, with GUA being the preferred binding partner. This can be explained by the presence of persistent hydrogen bonds (Table 3) whose strength is amplified in the low-dielectric environment. Both ASP and GLU form hydrogen bonds to N1 and N2 of GUA that are present 80 to 90% of the time, whereas the hydrogen bonds to CYT are only formed to a single atom, N4. The predominant geometric orientations (Figure 1) show that the negatively charged carboxyl groups of GLU and ASP form bidentate H-bonds to N1 and N2 of GUA, making this conformation particularly energetically favorable. Similar results are found for CYT with ASP and GLU (not shown), where bidentate hydrogen bonds are formed with N4 of CYT. In water simulations, these hydrogen bond patterns persist for a shorter period of time (Table 3), largely because the water molecules efficiently screen electrostatic interactions between nucleobases and sidechains.

**Table 3.** Percentage of time that a hydrogen bond is present in water and methanol simulations at the window around the minimum of the PMF as determined by the restraining distance *R*_0_

	ARG		LYSH		THR		SER		TYR		ASN		GLN		ASP		GLU	
	wat	met	wat	met	wat	met	wat	met	wat	met	wat	met	wat	met	wat	met	wat	met
GUA-N1	0	0	0	0	2	11	4	11	0	4	1	8	1	9	54	92	53	77
GUA-N2	0	0	0	0	3	9	4	10	0	4	3	11	2	11	32	79	33	88
GUA-O6	0	32	8	30	2	6	3	7	0	1	2	13	1	11	0	0	0	0
GUA-N7	0	28	7	24	2	4	2	3	0	1	3	10	2	4	0	0	0	0
CYT-O2	3	59	11	36	4	14	7	13	0	3	4	16	4	16	0	0	0	0
CYT-N3	2	35	5	17	3	6	3	1	0	1	5	17	3	17	0	0	0	0
CYT-N4	0	0	0	0	5	10	4	12	0	7	4	22	4	16	37	76	36	80
URA-N3	0	0	0	0	5	13	5	14	0	0	1	7	4	9	45	31	33	26
THY-N3	0	0	0	0	6	9	8	19	0	0	0	4	3	9	44	62	28	68

We only show hydrogen bonds that were present more than 20% of the time.

Finally, as potentially more relevant for realistic nucleic acid–protein complexes, we have also evaluated free energy differences between unbound nucleobases and sidechain analogs in water and their bound counterparts in methanol as the sum of the binding free energy in methanol (as shown in Table 1b) and the free energy of bringing the unbound state from water to methanol, Δ*G*_W−>M_(unb). The latter can be obtained through a procedure similar to the double decoupling method to calculate hydration free energies, where methanol simulations replace simulations in the gas phase. For charged amino-acid sidechains, the net charge of the system changes during the simulations and we apply corrections (41,50,51) to compensate for the approximate electrostatic treatment (see Supplementary Information for details). Importantly, inclusion of Δ*G*_W->M_(unb) leads to free energy differences which resemble the binding free energies in water significantly more than those in methanol (Supplementary Figure S2).

### Matching between mRNA composition and cognate proteins’ nucleobase affinity

**Figure 4.**
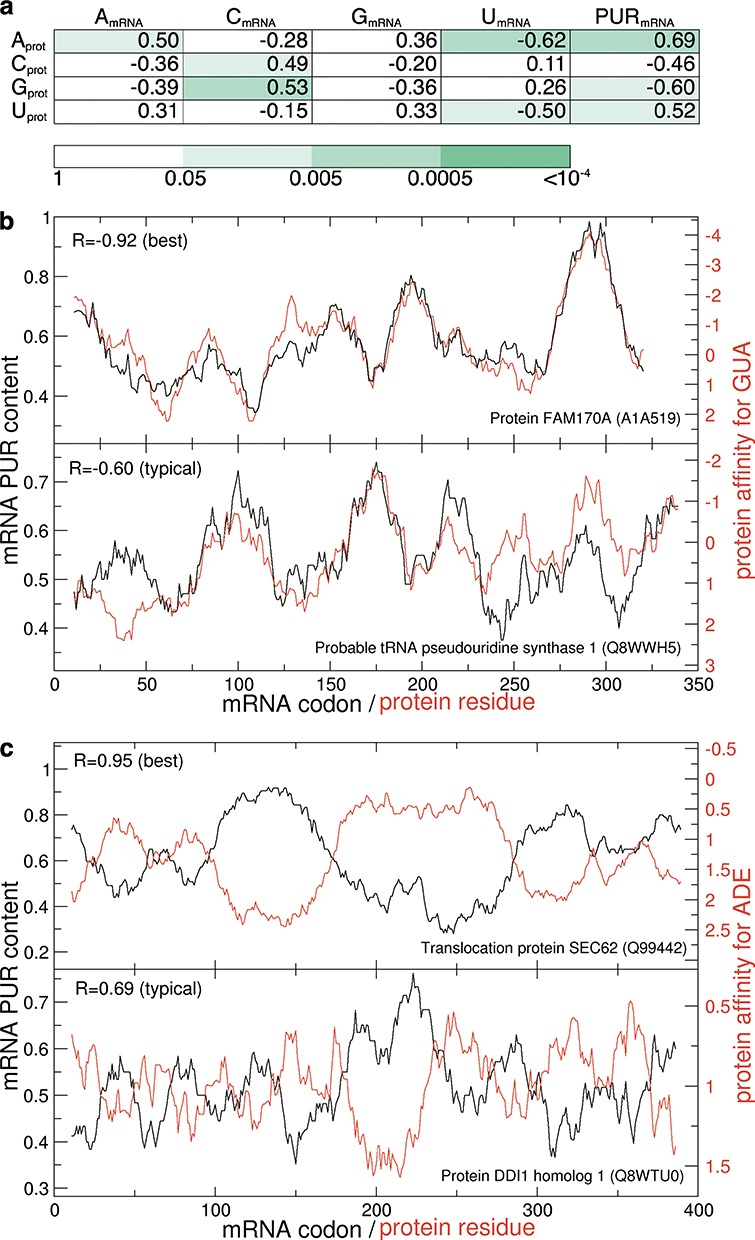
Correlations between mRNA PUR sequence profiles and cognate protein profiles of affinity for nucleobases in methanol. Median Pearson correlation coefficients with colors representing *P*-values obtained by shuffling of the affinity scales (**a**) and example profiles for GUA (**b**) and ADE (**c**) affinity are shown. The best and typical examples in (b) and (c) are chosen from proteins with a representative length (300–400 residues).

The binding free energies described above can be used to investigate the possibility of direct interactions between complete mRNA coding sequences and their cognate protein sequences as put forth in the mRNA-complementarity hypothesis (36–38) and to shed more light on the stereochemical hypothesis of the genetic code's origin (4–6). We have first compared window-averaged nucleobase density profiles of all annotated human mRNA coding sequences with window-averaged binding-affinity profiles of their cognate proteins by calculating Pearson *R*s between them. In Figure 4a, we summarize the results of this analysis for methanol scales by giving median Pearson correlation coefficients (*R*_median_) and the associated *P*-values over the entire human proteome. The strongest correlations are seen at the level of mRNA PUR density profiles and different protein preference profiles with A_prot_ versus PUR_mRNA_ leading the way with *R*_median_ = 0.69 (*P*-value = 0.001). However, statistically significant correlations or anti-correlations (*P*-value < 0.05) are also seen for A_prot_ versus A_mRNA_ (*R*_median_ = 0.50), C_prot_ versus C_mRNA_ (*R*_median_ = 0.49), U_prot_ versus U_mRNA_ (*R*_median_ = -0.50), A_prot_ versus U_mRNA_ (*R*_median_ = −0.62), G_prot_ versus C_mRNA_ (*R*_median_ = 0.53), G_prot_ versus PUR_mRNA_ (*R*_median_ = −0.60) and U_prot_ versus PUR_mRNA_ (*R*_median_ = 0.52). In Supplementary Figure S3 we show the distributions of *R*-values for the mRNA PUR density and all four RNA-nucleobase affinity profiles of their cognate proteins over the entire human proteome as evaluated using the methanol scale. In particular, G_prot_ and C_prot_ show a negative correlation with PUR density profiles (*R*_median_ = −0.60 and −0.46, respectively), while in the same context A_prot_ and U_prot_ exhibit a positive correlation (*R*_median_ = 0.69 and 0.52, respectively). In Figure 4b we show two examples of mRNA PUR-content and cognate protein G-affinity profiles as an illustration: one with the strongest correlation (top panel, ‘best’) and one with the correlation equal to *R*_median_ (*R* = −0.60) (lower panel, ‘typical’). As is evident, in the case of the best matching profile (FAM170A protein, *R* = −0.92) the mRNA PUR density can be predicted extremely well from the low-dielectric GUA affinity profile of its cognate protein: the regions in the protein sequence that show strong affinity for GUA are encoded by regions of codons that are rich in purines and vice versa. Furthermore, agreement between the two profiles remains remarkably good even for a typical, median protein in this regard (tRNA pseudouridine synthase I, *R* = −0.60). Overall, the strongest correlations can be found between methanol-based ADE-affinity on the side of proteins and PUR density on the side of mRNAs, as indicated in Supplementary Figure S3 by the narrowest peak and the largest *R*_median_ (0.69). The best and typical matching profiles are again shown (Figure 4c). The anti-correlation between the ADE affinity of the protein and the purine content of its cognate mRNA is strong even for a typical protein/mRNA pair: clearly, protein sequence stretches that show pronounced affinity for ADE are encoded by purine-poor regions and *vice versa*.

In contrast to methanol-based scales, water-based scales also exhibit strong levels of matching, but without any significant difference in specificity between different bases (Supplementary Figures S4 and Table S2). Concretely, PUR-content on the side of mRNAs is positively and indiscriminately correlated with affinity profiles for all four bases on the side of their cognate proteins, with URA-content mRNA profiles showing the strongest, yet still undifferentiated signal among the four individual bases (Supplementary Table S2). Finally, Supplementary Table S3 shows the *R*_median_ values for the pairwise combinations of nucleobase content profiles of mRNAs and nucleobase affinity profiles of their cognate proteins, where the nucleobase affinity is now based on Δ*G*_W,unb−>M,bound_. As expected, these correlations show similar trends to those obtained from binding free energies in water (Supplementary Table S2).

### Energetics of the complementarity hypothesis

**Figure 5.**
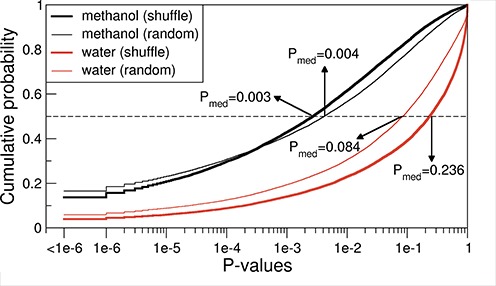
Cumulative probability distribution of *P*-values capturing for each human protein the fraction of 10^6^ randomized mRNAs with a lower ‘effective interaction energy’ with the protein sequence in question as compared to its original cognate mRNA as defined by water (red) or methanol (black) scales. Randomized mRNAs were generated either by shuffling the codons within mRNA sequences (thick lines) or by randomly picking codons from the genetic code table (thin lines). The median *P*-values over all human proteins are indicated with arrows.

In order to shed further light on the cognate mRNA–protein complementarity hypothesis, we have estimated the effective free energy of interaction for each cognate mRNA/protein pair in the human proteome by simply adding up free energies of interaction between each sidechain in a given protein sequence and the three nucleobases comprising its respective cognate codon, and then summing up such values over the whole sequence (here, glycine and proline contributions were set to zero on both sides). We have also evaluated the significance of such energies by calculating *P*-values that were defined as the fraction of 10^6^ randomized mRNAs that exhibit lower interaction free energies with a given protein than its cognate, native mRNA (see ‘Materials and Methods’ for details). Our analysis shows that the methanol scale appears to be strongly optimized for this with the median *P*-value of 0.003 over all human proteins, which is to be contrasted with the median *P*-value of 0.236 obtained using the water scale (Figure 5). In other words, a typical protein can pick out its native, cognate mRNA from a randomized mRNA sequence just based on their estimated energy of interaction in a low-dielectric environment with an error rate of only 3 in 1000. What is more, for ∼2300 proteins using the methanol scale and 600 proteins using the water scale, not one of the 10^6^ shuffled mRNAs exhibits lower interaction free energy than the cognate mRNA. Additionally, we have also generated randomized mRNA sequences by randomly picking codons at each position from a uniform distribution with each of the 53 non-stop, non-prolyl and non-glycine codons in the genetic code appearing with a probability of 1/53. The results of this procedure were very similar, with the median *P*-values of 0.004 for the methanol scale and 0.084 for the water scale (Figure 5). Finally, we have also recoded the mRNA sequences with the use of 10^3^ randomized genetic codes and for each protein determined interaction energies with their recoded cognate mRNAs. We found that interaction energies lower than those obtained using the original, universal genetic code can be found with a median *P*-value of 0.20 among the randomized genetic codes when using the methanol scale or 0.33 for the water scale.

### Binding free energies and the genetic code

How do the above results change if one focuses on individual codons and amino acids as suggested by the classical formulations of the stereochemical hypothesis? In Supplementary Table S4a (water scales) and 4b (methanol scales), we show the Pearson correlation coefficients between the affinity of amino acids for individual nucleobases and the average nucleobase content of their codons as present in the human proteome (see Supplementary Information for more details). In water, an appreciably strong relationship of this sort is seen only in the case of U_cdn_ and U_scl_ (*R* = −0.59, *P*-value = 0.01). However, negative Pearson correlation coefficients are also obtained between U_cdn_ and all three other nucleobase affinity scales (Supplementary Table S4a). In methanol, on the other hand, G_cdn_ and U_cdn_ exhibit weak to moderate anti-correlation with the respective G_scl_ and U_scl_ scales, with *R* = −0.37 (*P*-value = 0.14) and *R* = −0.63 (*P*-value = 0.005), respectively, while the opposite trend is observed for ADE (*R* = 0.54, *P*-value = 0.02) and CYT (*R* = 0.42, *P*-value = 0.08) in methanol (Supplementary Table S4b).

Finally, we have also analyzed the effective interaction energy of the genetic code table as defined through the sum of interaction energies between all codons in the table (excluding stop, GLY and PRO codons) and their cognate sidechains, whereby sidechain interaction energy with a given codon was taken as the sum of the interaction energies of its constituent nucleobases. By generating 10^6^ randomized codes, determining their effective interaction energies and comparing them with the energy of the native, universal genetic code, we could determine the *P*-values capturing the significance of optimization of the native code when it comes to codon/sidechain interaction energies. Using the binding free energies obtained from the simulations in methanol, the obtained *P*-value was equal to 0.14 if weighting with codon frequencies in the human proteome was used or 0.09 with no weighting. When using the binding free energies in water, *P*-values of 0.27 (with weighting) or 0.60 (without weighting) were obtained.

## DISCUSSION

Despite the fundamental importance of protein-nucleic acid interactions in all known biological systems, this is to the best of our knowledge the first time that the absolute binding free energies for all combinations of standard nucleobases and amino-acid sidechain analogs have been evaluated within a single self-consistent framework. While the calculated binding free energies do strongly depend on the chosen MD force field, we believe that the GROMOS force field 54a8 is particularly suitable for such calculations as it was explicitly parameterized to match the highly relevant thermodynamic properties of amino-acid sidechain analogues including hydration free energies (41). Although the GROMOS nucleobase parameters are less accurate in this regard (52), the favorable comparison between our binding free energies and the extant experimental data (Figure 3) suggests that this may be less critical. Overall, with water as a solvent, all nucleobases bind preferentially to hydrophobic sidechains and in particular to the aromatic ones. In low-dielectric methanol, on the other hand, GUA and CYT bind most strongly to the negatively charged ASP and GLU, while these sidechains are the least favorable binders for THY and URA. The majority of the differences in binding preferences in the two environments can be explained by: (i) the destabilizing effect of the low-dielectric environment on stacking interactions and (ii) the fact that in bulk water electrostatic interactions between nucleobases and sidechains are significantly screened. We believe that these two basic principles, which have also been described in different guises before (48,49), constitute the foundation for understanding specificity in nucleic acid–protein interactions from the perspective of individual nucleobases and amino-acid sidechains.

In addition to matching the experimental data available for analogous systems as discussed above, the binding free energies determined herein exhibit a good agreement with structural and energetic analyses of nucleic-acid/protein complexes and their binding interfaces. Multiple research groups have analyzed interactions at protein–RNA/DNA interfaces as seen in high-resolution 3D structures (7,9–12,16,24). In protein–DNA complexes in particular, most hydrogen bonds form with the Hoogsteen edge of nucleobases since the Watson–Crick edges are typically unavailable due to base pairing. More specifically, interactions with at least two hydrogen bonds are found predominantly between GUA/ARG, LYSH and ADE/ASN, GLN (11,24). The GUA/ARG interactions via the Hoogsteen edge feature prominently at protein–RNA interfaces as well, although a significant presence of GUA/ASP,GLU pairs via the Watson–Crick edge is also observed (7,53). In our simulations in methanol, we also find significant interactions of ASP and GLU at the Watson–Crick edge of GUA, but the preferences of ARG and LYSH for GUA are not as pronounced (Tables 2 and 3). This is probably due to the fact that in our simulations we do not include the negatively charged phosphate backbone which is chiefly responsible for attracting the positively charged amino-acid sidechains. In a related analysis of 3D structures of nucleotide–protein complexes, it was found that most interactions for ADE and URA were with the protein backbone, whereas for GUA this was ASP with hydrogen bonds at the Watson–Crick edge (10). The latter again corresponds very well with our simulations in methanol (Tables 2 and 3 and Figure 1). Finally, the present results qualitatively agree with the knowledge-based potentials recently derived from a large set of structures of RNA–protein complexes by us (37). In particular, strong favorable interactions between GUA and GLU were seen there as well.

Throughout this study, we have used amino-acid sidechain analogs defined by replacing the Cα atom by a hydrogen atom. This approach is not applicable to glycine, where one would be left with a hydrogen molecule and proline, a secondary amine. While glycine remains outside the reach of the present approach, proline sidechain can be represented by cyclopentane (CPE). In this model, the atomic charges are equal to zero and the bond, angle and dihedral angle parameters are equal to the parameters for the carbon atoms in the proline sidechain. We have applied the same computational framework to CPE as to other sidechain analogs, and have obtained the binding free energies of −2.7, −1.9, −2.8, −1.8 and −2.5 kJ/mol in water and −0.3, 2.1, 1.4, 0.0 and 2.7 kJ/mol in methanol for ADE, CYT, GUA, URA and THY, respectively. Importantly, including these values as proxies for proline binding free energies in the comparison of mRNA/protein composition profiles leads to only minor changes in the median Pearson *R*-values, with most correlations and anti-correlations actually becoming stronger in absolute value. For example, the median Pearson correlation coefficients between the mRNA PUR profiles and their cognate proteins nucleobase-affinity profiles in methanol change from 0.69 to 0.73 (ADE), −0.46 to −0.52 (CYT), −0.60 to −0.61 (GUA) and 0.52 to 0.56 (URA), and similar effects are seen in water. On the other hand, the effective free energies of interaction between cognate mRNA/protein pairs become less significant if the CPE results are included (the *P*-value of cognate interactions as compared to those with randomized mRNAs in methanol changes from 0.003 to 0.06). However, as CPE has two additional methyl groups as compared to the *bona fide* proline sidechain, and is therefore a fundamentally different kind of a sidechain analog as compared to other groups in our study, both of these results should be treated with caution.

Although fundamentally reductionist in nature, the derived binding free energies have helped us demonstrate a remarkable relationship between nucleobase density profiles of natural mRNA coding sequences and nucleobase affinity profiles of their cognate protein sequences, in close agreement with previous knowledge-based results and the mRNA–protein complementarity hypothesis (37). Indeed, one obtains stronger signatures of complementary binding if one examines complete mRNA and protein sequences rather than individual sidechains and their respective codons (36–38). Moreover, the low-dielectric scales of the two purine bases act antagonistically in that protein GUA-affinity profiles match mRNA PUR-density profiles, while ADE-affinity profiles exhibit the opposite behavior. Finally, protein URA-affinity profiles obtained using both water and methanol scales as well as protein CYT-affinity profiles obtained using the water scale match mRNA-PYR density profiles, agreeing with our previous PR-scale based results (36).

On the other hand, the behavior of the CYT low-dielectric scale goes against what was seen with its knowledge-based counterpart and is antithetical to both stereochemical hypothesis and cognate mRNA–protein complementarity hypothesis. In addition to potential force-field inaccuracies, a part of the explanation may lie in the fact that, unlike the absolute scales obtained herein, the knowledge-based scales are intrinsically relative. If one, for example, examines the relative GUA–CYT binding free energy scale, the qualitative agreement with the knowledge-based results is recovered (results not shown). Alternatively, it is possible that methanol's dielectric constant may be too low to accurately mimic realistic nucleic acid–protein interfaces when it comes to CYT-based interactions and that in this case water-based free energies may be more appropriate. Uncertainties with CYT-scales notwithstanding, the opposite behavior of GUA and ADE scales, which has now been confirmed using multiple approaches, suggests that the genetic code may have been shaped by positive binding interactions in some cases, but also their active avoidance in other cases (38,54). Namely, a high ADE content in a given mRNA may be there to weaken any putative complementary binding with a cognate protein, providing an important modification of the complementarity hypothesis as was also discussed before (38). Still, primarily due to the dominant contribution of GUA-based interactions, the estimated total in-frame binding energy between mRNAs and their cognate proteins is significantly lower than that obtained with randomized mRNA sequences, speaking again in favor of the complementarity hypothesis. While only future work can provide a definitive assessment of this exciting possibility, we see its potential to affect all contexts where nucleic acid/protein interactions are of relevance as undeniable (38,55,56). Importantly, regarding the origin of the coding relationship between proteins and mRNAs, the present results support the possibility that certain mRNAs, depending on their composition, could have served as direct templates for the synthesis of proteins, but that the reverse may also be true. Of course, in this scenario, the coding in both directions would have been fuzzy: the same mRNAs would have coded for multiple proteins with similar nucleobase-binding propensity profiles, but also the same protein would have coded for multiple mRNAs with similar nucleobase density profiles.

Going beyond just the mRNA/protein relationship, however, the absolute binding free energies between all standard nucleobases and amino-acid sidechains presented herein provide a comprehensive, quantitative alphabet for understanding the thermodynamics of nucleic acid/protein interactions and it is our hope that they will find usage in both fundamental studies of nucleic acid/protein biology and various practical applications such as docking, interface design and structure prediction.

## SUPPLEMENTARY DATA

Supplementary Data are available at NAR Online.

SUPPLEMENTARY DATA
