# Supplementary material for: Absolute binding-free energies between standard RNA/DNA nucleobases and amino-acid sidechain analogs in different environments
Source: Nucleic Acids Res. 2014 Dec 30;43(2):708–18. doi: 10.1093/nar/gku1344 (PMC4333394; doi:10.1093/nar/gku1344)
Supplement: SUPPLEMENTARY DATA [file supp_43_2_708__index.html]

Absolute binding-free energies between standard RNA/DNA nucleobases and amino-acid sidechain analogs in different environments — Absolute binding-free energies between standard RNA/DNA nucleobases and amino-acid sidechain analogs in different environments — SUPPLEMENTARY DATA 

# Absolute binding-free energies between standard RNA/DNA nucleobases and amino-acid sidechain analogs in different environments

## SUPPLEMENTARY DATA

**Files in this Data Supplement:**

- SUPPLEMENTARY DATA
