# Supplementary material for: Absolute binding-free energies between standard RNA/DNA nucleobases and amino-acid sidechain analogs in different environments
Source: Nucleic Acids Res. 2014 Dec 30;43(2):708–18. doi: 10.1093/nar/gku1344 (PMC4333394; doi:10.1093/nar/gku1344)
Supplement: SUPPLEMENTARY DATA [file supp_gku1344_nar-02878-f-2014-File010.pdf]

submitted to Nucleic Acids Research

Absolute binding free energies between standard RNA/DNA nucleobases and amino-acid sidechain analogs in different environments

Anita de Ruiter & Bojan Zagrovic*

Department of Structural and Computational Biology, Max F. Perutz Laboratories, University of Vienna, Vienna 1030, Austria

December 2014

Supplementary Information

Index

**Figure S1**	Potential of mean force (PMF) curves for the binding between ADE, CYT, URA and THY and all side chains in water and in methanol	**3**
**Figure S2**	Free energy of bringing side chains and nucleobases from water to methanol and of bringing each pair of side chain and nucleobase from the unbound state in water to the bound state in methanol	**4**
**Figure S3**	Distributions of correlations between mRNA PUR content and protein affinity for nucleobases in methanol	**5**
**Figure S4**	Correlations between mRNA PUR content and protein affinity for nucleobases in water	**6**
**Table S1**	Correlations between binding free energy scales	**7**
**Table S2**	Median values of pairwise Pearson coefficients between sequence profiles of mRNA nucleobase content and their cognate proteins' profiles of binding free energy with a given nucleobase in water	**8**
**Table S3**	Median values of pairwise Pearson coefficients between sequence profiles of mRNA nucleobase content and their cognate proteins' profiles of binding free energy with a given nucleobase in methanol including $\Delta G_{W\text{->}M}$(unb)	**9**
**Table S4**	Pairwise Pearson coefficients between binding free energies of side chains to nucleobases and average nucleobase codon content over the whole human proteome in water and methanol	**10**
**Theory**	Theory section explaining how the PMF, the binding free energies and the free energy difference of bringing the unbound state from water to methanol are calculated	**11**
**Methods**	Umbrella sampling and analysis of the matching between mRNA composition and cognate proteins' nucleobase affinity	**16**

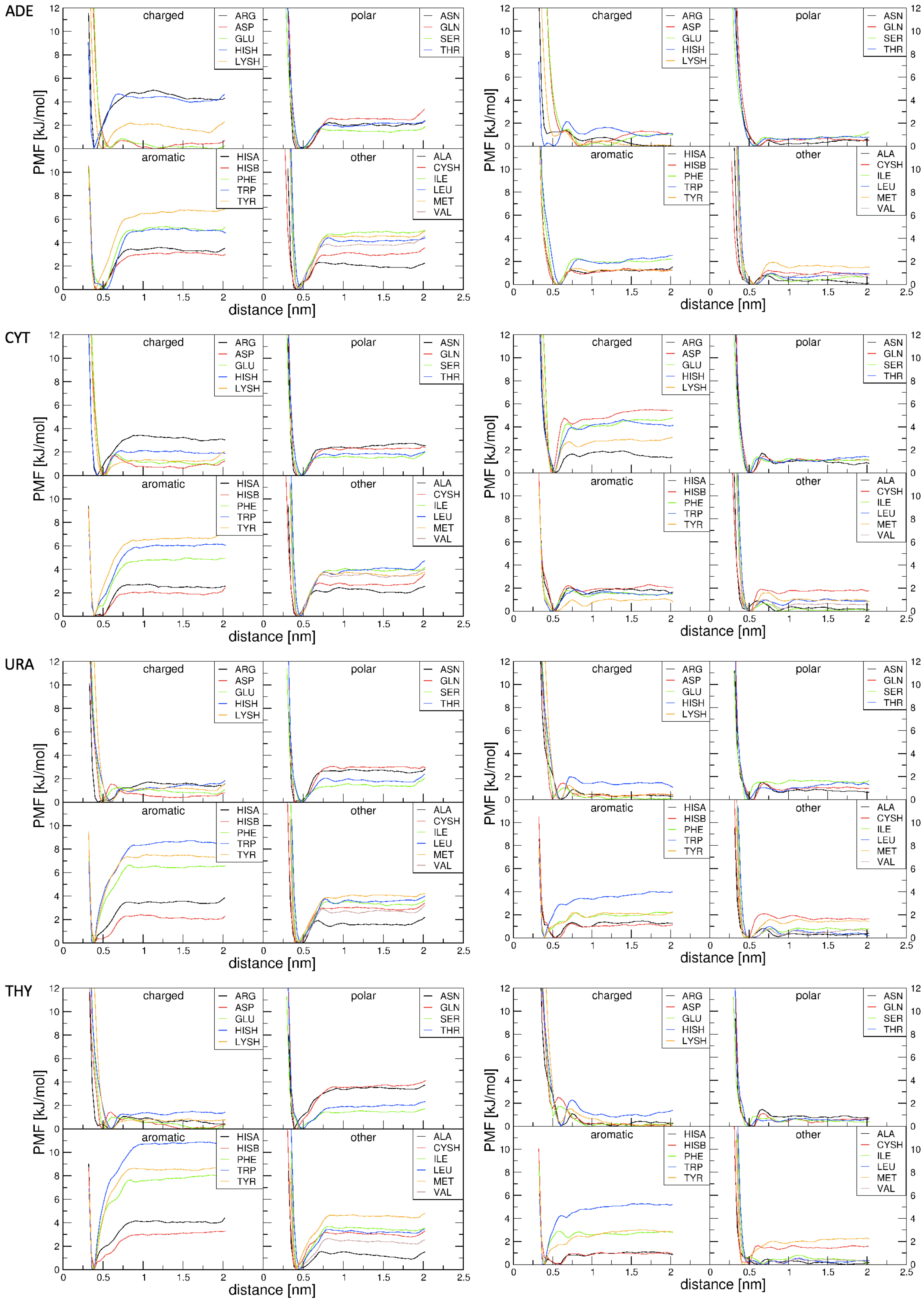

Figure S1. Potential of mean force (PMF) curves for ADE, CYT, URA and THY (rows) with all side chains in water (left) and in methanol (right).

A

$\Delta G^{res}_{W\to M}(unb)$	
ALA	-6.28
ARG	-21.31
ASN	-8.60
ASP	23.79
CYSH	-8.08
GLN	-9.87
GLU	22.79
HISA	-6.76
HISB	-2.72
HISH	-13.67
ILE	-15.47
LEU	-15.69
LYSH	-18.64
MET	-9.74
PHE	-16.12
SER	-2.95
THR	-4.99
TRP	-20.00
TYR	-14.76
VAL	-12.74

B

$\Delta G^{base}_{W\to M}(unb)$	
ADE	-17.26
CYT	-12.10
GUA	-13.57
URA	-12.12
THY	-14.67

C

	A	C	G	U	T
ALA	-22.3	-15.9	-16.8	-17.1	-20.0
ARG	-35.8	-32.6	-36.5	-32.3	-35.1
ASN	-25.4	-19.6	-20.2	-19.3	-22.0
ASP	8.3	9.8	3.8	14.6	14.4
CYSH	-24.9	-19.4	-19.9	-19.1	-21.3
GLN	-26.4	-21.2	-22.1	-21.2	-23.2
GLU	7.8	9.1	2.2	13.9	12.8
HISA	-23.8	-18.7	-20.8	-18.7	-20.9
HISB	-19.5	-14.7	-16.5	-14.8	-17.3
HISH	-30.1	-27.5	-29.1	-25.3	-27.3
ILE	-32.0	-26.3	-27.8	-27.9	-30.1
LEU	-32.9	-27.6	-27.5	-27.0	-30.3
LYSH	-32.9	-30.8	-33.2	-29.1	-31.1
MET	-27.3	-20.7	-24.3	-22.2	-25.3
PHE	-34.0	-27.4	-29.8	-28.9	-29.0
SER	-19.2	-12.7	-13.3	-13.0	-14.6
THR	-21.9	-15.3	-16.0	-15.7	-16.8
TRP	-38.5	-31.9	-34.1	-32.0	-35.6
TYR	-31.9	-27.1	-28.4	-27.5	-30.4
VAL	-29.9	-23.6	-24.4	-24.9	-27.4

Figure S2. Free energy of bringing amino-acid side chains (A) and nucleobases (B) from water to methanol and total free energy differences of bringing each pair of amino-acid side chains and nucleobases from the unbound state in water to the bound state in methanol (C).

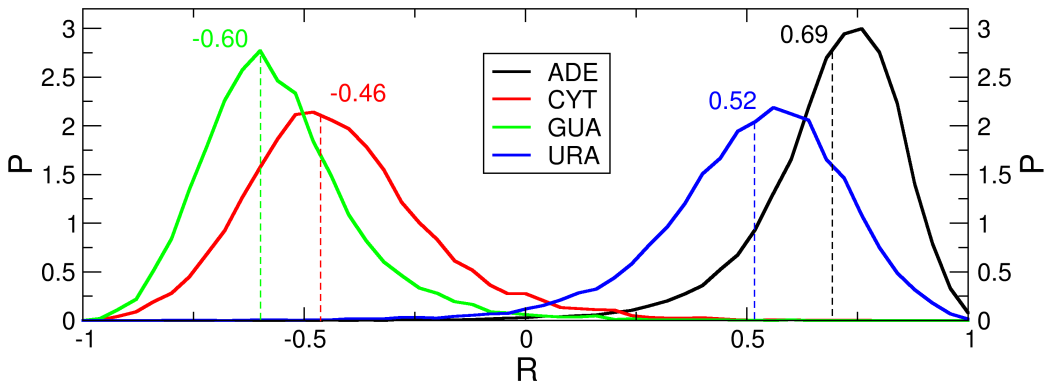

Figure S3. Distributions of the Pearson correlation coefficients between mRNA PUR sequence profiles and cognate protein profiles of affinity for nucleobases in methanol.

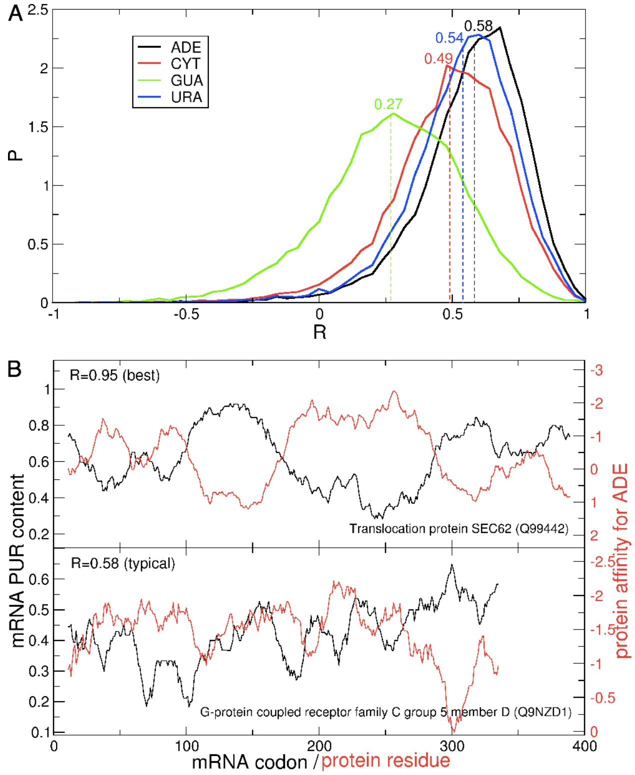

Figure S4. Correlations between mRNA PUR content and protein affinity profiles for nucleobases in water. Distributions of correlation coefficients (A) and exemplary profiles for ADE affinity (B) are shown. The best and typical matches for the examples in B and C are chosen from proteins with a representative length (300-400 residues).

A

water		A	C	G	U	T
water	A	1.00	0.94	0.89	0.94	0.92
	C	0.94	1.00	0.92	0.95	0.90
	G	0.89	0.92	1.00	0.89	0.84
	U	0.94	0.95	0.89	1.00	0.98
	T	0.92	0.90	0.84	0.98	1.00

B

methanol		A	C	G	U	T
water	A	0.79	-0.18	-0.34	0.88	0.78
	C	0.71	-0.16	-0.29	0.84	0.80
	G	0.61	0.09	-0.06	0.78	0.70
	U	0.77	-0.29	-0.42	0.83	0.80
	T	0.81	-0.34	-0.49	0.83	0.78

C

water		A	C	G	U	T
methanol	A	1.00	-0.24	-0.40	0.67	0.57
	C	-0.24	1.00	0.85	-0.25	-0.30
	G	-0.40	0.85	1.00	-0.41	-0.48
	U	0.67	-0.25	-0.41	1.00	0.87
	T	0.57	-0.30	-0.48	0.87	1.00

Table S1. Pearson correlation coefficients between the binding free energy scales. A: water vs. water, B: water vs. methanol, C: methanol vs. methanol.

	A_{mRNA}	C_{mRNA}	G_{mRNA}	U_{mRNA}	PUR_{mRNA}
A_{prot}	0.34	-0.18	0.39	-0.56	0.58
C_{prot}	0.35	-0.10	0.25	-0.52	0.49
G_{prot}	0.11	0.10	0.23	-0.43	0.27
U_{prot}	0.33	-0.19	0.34	-0.50	0.54

1 0.05 0.005 0.0005 $<10^{-4}$

Table S2. Median values of pairwise Pearson coefficients between sequence profiles of mRNA nucleobase content and their cognate proteins' profiles of binding free energy with a given nucleobase in water. Colors represent the P-values, obtained by shuffling the affinity scales 10^4 times.

	A_{mRNA}	C_{mRNA}	G_{mRNA}	U_{mRNA}	PUR_{mRNA}
A_{prot}	0.22	-0.23	0.29	-0.28	0.40
C_{prot}	0.16	-0.17	0.25	-0.23	0.32
G_{prot}	0.10	-0.09	0.20	-0.20	0.24
U_{prot}	0.20	-0.21	0.27	-0.26	0.37

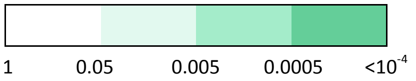

Table S3. Median values of pairwise Pearson coefficients between sequence profiles of mRNA nucleobase content and their cognate proteins' profiles of binding free energy with a given nucleobase in methanol including $\Delta G_{W->M}$(unb). Colors represent the P-values, obtained by shuffling the affinity scales 10^4 times.

A

	A_{cdn}	C_{cdn}	G_{cdn}	U_{cdn}	PUR_{cdn}
A_{scl}	0.40	0.08	0.21	-0.67	0.54
C_{scl}	0.41	0.18	0.09	-0.64	0.45
G_{scl}	0.26	0.29	0.08	-0.56	0.30
U_{scl}	0.40	0.12	0.10	-0.59	0.44

1 0.05 0.005 0.0005 $<10^{-4}$

B

	A_{cdn}	C_{cdn}	G_{cdn}	U_{cdn}	PUR_{cdn}
A_{scl}	0.54	-0.02	0.14	-0.67	0.61
C_{scl}	-0.25	0.42	-0.20	0.14	-0.40
G_{scl}	-0.25	0.47	-0.37	0.26	-0.53
U_{scl}	0.34	0.03	0.27	-0.63	0.54

1 0.05 0.005 0.0005 $<10^{-4}$

Table S4. Pairwise Pearson correlation coefficients between binding free energies of amino acids to a given nucleobase (scl) and the average nucleobase content of their codons over the whole human proteome (cdn). Results are shown for water (A) and methanol (B); colors represent the P-values, obtained by shuffling the affinity scales 10^6 times.

Theory

Potentials of mean force and binding free energy

The potential of mean force (PMF) is defined as the free energy along a reaction coordinate *r*, where *r* represents e.g. a specific angle or a distance. A prerequisite for the calculation of a PMF is sufficient sampling at each value of *r*. Unfortunately, this also includes some higher energy configurations that are, by definition, sampled only rarely in molecular dynamics (MD) simulations. Visiting such configurations in simulation can be enhanced by applying umbrella sampling (US), which makes use of biasing potentials to overcome energy barriers. The biasing potentials in this study are defined as harmonic distance restraints centered at several values along the reaction coordinate *r*. Although the performed simulations give rise to biased probabilities along *r* for each window separately, the weighted histogram analysis method (WHAM) is used to combine and unbias these results, leading to a raw potential of mean force, $\Delta G_{WHAM}(r)$. This can in turn be used to obtain the probability $P(r)$ to find the two molecules at a radial distance *r*.

$$P(r) = C_1 e^{-\Delta G_{WHAM}(r)/k_B T} \qquad 1$$

Here, C_1 is a constant, k_B is the Boltzmann constant and *T* the temperature in the units of Kelvin. The PMF is defined in terms of the radial distribution function $g(r)$:

$$\Delta G_{PMF}(r) = -k_B T \, ln \, g(r) \qquad 2$$

The radial distribution function based on MD simulations is defined as the ratio between $P(r)$ and the probability of finding the two molecules at distance *r* in the case of a homogenous distribution, as show in eq. 3.

$$g(r) = \frac{P(r) V_{box}}{V(r)} \qquad 3$$

Here, V_{box} is the volume of the box and $V(r)$ the volume available at distance *r*. This definition of $g(r)$ includes the correction for the fact that the available volume increases with *r*, i.e. includes the Jacobian which accounts for the transformation of Cartesian positions of the molecules to intermolecular distances. The PMF can thus be obtained by combining equations 1-3:

$$\begin{aligned}\Delta G_{PMF} &= -k_B T \, ln \, P(r) - k_B T \, ln \, V_{box} + k_B T \, ln \, 4\pi r^2 dr \\ &= \Delta G_{WHAM}(r) + 2 k_B T \, ln \, r + C_2\end{aligned} \qquad 4$$

The PMF can also be used to calculate the free energy of binding, with a necessary prerequisite being that several corrections be applied. For this, two regions along the reaction coordinate *r* are defined, V_b and V_{unb} which correspond to bound and unbound states, respectively. The lower boundary of V_b is simply defined as the minimum distance for which $\Delta G_{WHAM}(r)$ is determined. For the upper boundary of V_b, the distance for which $\Delta G_{WHAM}(r)$ shows a maximum value is chosen. The unbound region V_{unb} is defined as the region between *x* and *y*, where *x* and *y* are chosen such, that $\Delta G_{PMF}(r)$ is flat in this whole region based on visual inspection. The concentration penalty as shown in eq. 5 accounts for the free energy associated with bringing the ligand from V_{unb}, to the volume available at *r*.

5

$$\Delta G_{conc}(r) = -k_B T \, ln \frac{V(r)}{V_{unb}} = -k_B T \, ln \frac{4\pi r^2 dr}{V_{unb}}$$

Here, V_{unb} is the volume available in the unbound state during the simulations. It can easily be shown that, apart from a constant, the sum of this penalty and ΔG_{PMF} is the same as ΔG_{WHAM}. The free energy difference between the bound and unbound state can now be calculated using:

$$\begin{aligned} \Delta G(V_b, V_{unb}) &= -k_B T \, ln \int_{V_b} e^{-\Delta G_{WHAM}(r)/k_B T} dr + k_B T \, ln \int_{V_{unb}} e^{-\Delta G_{WHAM}(r)/k_B T} dr \\ &= -k_B T \, ln \int_{V_b} 4\pi r^2 e^{-\Delta G_{PMF}(r)/k_B T} dr + k_B T \, ln \int_{V_{unb}} 4\pi r^2 e^{-\Delta G_{PMF}(r)/k_B T} dr \end{aligned} \quad (6)$$

The definition used here for the unbound state is not very general, as it depends on the largest simulated distance. For this reason, a standard state correction is added to $\Delta G(V_b, V_{unb})$, making the free energy independent of the choice of the unbound state.

$$\Delta G_{std} = -k_B T \, ln \frac{V_{unb}}{V_0} \quad (7)$$

Here, V_0 is the standard volume of 1.661 nm^3. Although the free energy difference is hereby independent on the exact choice of the unbound state, it is still slightly dependent on the definition of the bound state.

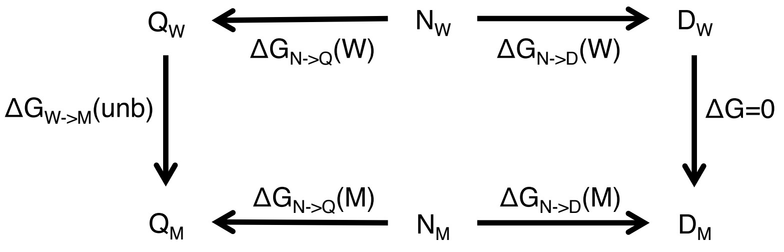

Scheme S1. Thermodynamic cycle for the calculation of the free energy difference between a molecule Q in water and in methanol. Subscripts W and M refer to the solvents water and methanol, D to the dummy state without partial charges and no Lennard-Jones interactions with the surroundings, N to the neutral state where all partial charges are zero, Q to the state with partial charges.

Free energy from unbound (water) to bound (methanol) state

The free energy of bringing the unbound state from water to methanol, $\Delta G_{W\text{->}M}(unb)$ can be obtained from the thermodynamic cycle as shown in scheme S1, where the subscripts W and M denote the solvents water and methanol, respectively, Q denotes a molecule with partial charges, N denotes the molecule with its partial charges set to zero and D denotes a dummy state, where the partial charges and Lennard-Jones interactions of all atoms of the molecule are set to zero, leaving only covalently bound dummy atoms.

The free energy difference between D_W and D_M is 0, because neither of these states interacts with the solvent. We thus only have to determine the free energy difference between D and Q in both solvents. Since the distance between the sidechain analog and nucleobase is larger than the cutoff of electrostatic interactions in the unbound state, we assume that their contributions to $\Delta G_{W->M}$(unb) can be calculated from separate simulations. The thermodynamic cycle of scheme S1 is thus calculated separately for individual nucleobases as well as individual amino-acid side chains.

The total free energy of changing a neutral, dummy molecule D into a charged, fully interacting molecule Q can be calculated with:

$$\Delta G_{D->Q} = \Delta G_{D->N} + \Delta G_{N->Q} + \Delta G_{std} \quad \textbf{8}$$

where ΔG_{std} is the standard state correction.

The raw charging free energy (ΔG_{raw}) is obtained from TI simulations with 11 equally distributed λ-states between 0 and 1, where λ=0 represents the neutral state and λ=1 the fully charged state. Simulations are 1 ns in length at each λ-state. Integration over $\langle dH/d\lambda \rangle_\lambda$ using the trapezoidal rule results in ΔG_{raw}. In order to obtain a method-independent charging free energy, several effects must be accounted for. The charging free energies were obtained from simulations with cutoff truncation, a reaction field correction and periodic boundary conditions, which requires the following correction terms (1–3);

A. The type A correction accounts for the error in the solvent polarization caused by the use of effective electrostatic interactions, rather than Coulombic interactions. It can be divided into two parts, where the first one compensates for the neglected interactions that were outside of the cutoff sphere (A_1) and the second one compensates for the errors made within the cutoff sphere (A_2).
B. The type B correction compensates for the error in the solvent polarization due to the finite microscopic size of the system and the periodicity.
C. The type C correction accounts for the error in the potential at the ionic site due to an inappropriate summation scheme (C1).
D. The type D correction compensates for the inaccurate dielectric permittivity of the solvent model.

The formulas for the corrections described below are appropriate for simulations with a non-polarizable force field, periodic boundary conditions (PBC) with cubic boxes, electrostatics with cutoff truncation (CT) and Barker-Watts (BW) reaction field correction with a molecular-based cutoff (BM).

Correction **A1** can be calculated with:

$$\Delta \mathrm{G}_{\mathrm{A}^1}^{\mathrm{CT}} = -(8\pi\varepsilon_0)^{-1}\mathrm{N}_\mathrm{A}\mathrm{q}_I^2(1\text{-}\varepsilon_s^{'-1})\mathrm{R}_C^{-1} \quad \textbf{9}$$

where ε_0 is the permittivity of vacuum, q_I the net charge of the 'ion', $\varepsilon_s^{'}$ is the permittivity of the solvent model (61.0 for SPC water and 18.6 for methanol) and R_C the cutoff radius.
The definition of the $\mathbf{A_2}$ correction term is based on continuum-electrostatics calculations, but this numerical procedure can be substituted by an empirical expression as proposed by Reif & Hünenberger (eq 43. in ref (2)), with virtually no loss of accuracy. The empirical function used is:

$$\Delta G_{A^2}^{BW} = \left(8\pi\varepsilon_0\right)^{-1} N_A q_I^2 R_C^{-1} \Big\{ \text{-}10^{-1}\text{b}_1 \Big[1\text{-b}_2\varepsilon_s^{'-1} - \text{b}_3\varepsilon_\text{s}^{'\text{-}2} + \text{b}_4 \left(\varepsilon_\text{s}^{'2} \left(1 + 10^{\text{-}1}\text{b}_5\varepsilon_\text{s}^{'} + 10^{-2} b_6 \varepsilon_\text{s}^{'2} \right) \right)^{-1} \Big] + \text{b}_7 \left(\text{R}_C^{-1}\text{R}_\text{I} \right)^3 \left(1\text{-b}_8\varepsilon_s^{'-1} + \text{b}_9\varepsilon_\text{s}^{'\text{-}2} + 10^{\text{-}1}\text{b}_{10}\varepsilon_\text{s}^{'} \right) \left(\varepsilon_\text{s}^{'} + \text{b}_{11} \right)^{\text{-}1} \Big\} \quad \mathbf{10}$$

where the optimized fitting coefficients bj, j=1…11 can be found in ref (2) and R_I represents the ionic radius. The ionic volume is estimated by measuring the difference in box volume between the neutral state and the dummy state. The ion is assumed to be spherical and R_I can thus be determined from $V_I = 4/3\ \pi\ R_I^3$.

The type **B** correction term is related to the type A corrections and can be expressed as:

$$\Delta G_B^{CT} = -\frac{R_I}{R_C} \exp\left[\mu^{CT}\left(\varepsilon_s^{'}\right) \frac{L}{2R_C} + v^{CT}\left(\varepsilon_s^{'}\right) \right] \times \left[\left(8\pi\varepsilon_0\right)^{-1} N_A q_I^2 \left(1 - \varepsilon_s^{'-1}\right) R_I^{-1} + \Delta G_A^{CT} \right] \quad \mathbf{11}$$

where μ^{CT} and v^{CT} can again be determined from empirical expression as proposed by ref (2)

$$\mu^{BW}\left(\varepsilon_s^{'}\right) = d_{\mu,1} + d_{\mu,2}\varepsilon_s^{'-1} \quad \mathbf{12}$$

$$v^{BW}\left(\varepsilon_s^{'}\right) = d_{v,1} + d_{v,2}\varepsilon_s^{'-1}$$

with $d_{\mu,i}$ and $d_{v,i}$, i=1,2 representing the fitting coefficients as stated in ref (2).

The **C1** correction term is calculated analytically with:

$$\Delta G_{C1}^{BM} = -N_A \left(6\varepsilon_0\right)^{-1} \frac{2\left(\varepsilon_{BW} - 1\right)}{2\varepsilon_{BW} + 1} N_S \gamma_S^{'} q_I \frac{1 - \left[\frac{4}{3}\pi R_C^3 \right]^{-1} V_I}{\langle L \rangle^3 - V_I} \quad \mathbf{13}$$

where ε_{BW} is the permittivity assigned to the medium outside of the cutoff sphere (which is ideally set to permittivity of the solvent model ε'_s), Ns is the number of solvent molecules, γ'_s is the quadropole moment trace of the solvent model and L is the box length. Assuming that the solvent has a single van der Waals interaction site (which is its molecular center M) and that it is spherical, γ'_s can be calculated through the analytical function:

$$\gamma_S^{'} = \sum_i^N q_i r_i^2 \quad \mathbf{14}$$

where N is the number of atoms in the solvent molecule, q_i is the partial charge of atoms i of the solvent and r_i is the distance of atom i to the center M. The above mentioned assumptions are valid for the SPC water model and applying eq. 14 results in γ'_s=0.0082e·nm^2. In order to be able

to calculate the C1 correction term for methanol, we assume that the oxygen atom is the molecular center of methanol, that methanol looks spherical and rotates isotropically around the oxygen, even though the carbon atom also is a van der Waals interaction site. The effective quadropole moment trace calculated with eq. 14 is then equal to 0.0103$e \cdot nm^2$. To test if these assumptions are reasonable for methanol, the charging free energies for ASP in methanol are calculated, once from simulations with ε_{BW} equal to 18.6 (BM scheme) and once without reaction field correction beyond the cutoff sphere (ε_{BW}=1, CM scheme). In the first case we use the effective quadropole moment trace to calculate the C1 correction and in the latter case, no C1 correction term is required (as can be easily seen from eq. 14). After applying all corrections, the difference between the BM and CM scheme was only around 2.5 kJ/mol (results not shown). Taking into account that CM calculations are very crude because of the large cutoff artifacts, we can say that the effective quadropole moment trace of 0.0103 $e \cdot nm^2$ is able to account for the exclusion potential of methanol very well and we used this value in all further calculations.

The **D** type correction is calculated with:

$$\Delta G_D = \left(8\pi\varepsilon_0\right)^{-1} N_A q_I^2 \left(\varepsilon_s^{-1} - \varepsilon_s^{'-1}\right) R_I^{-1} \qquad \textbf{15}$$

where ε_s is the experimentally determined permittivity of the solvent. Here we have used ε_s =78.4 for water and ε_s =33.0 for methanol.

The final charging free energy is thus determined by:

$$\Delta G_{N->Q} = \Delta G_{raw} + \Delta G_{A1} + \Delta G_{A2} + \Delta G_B + \Delta G_{C1} + \Delta G_D \qquad \textbf{16}$$

Note that the above corrections all scale with the net charge and thus will only contribute to the charging free energies of the charged amino acid side chains and not to the neutral amino acid side chains or nucleobases.

The free energy of cavity formation, $\Delta G_{D->N}$, is again calculated from TI simulations, now with 22 λ-states between 0 and 1, where λ=0 represents the neutral state (all atomic partial charges set to zero) and λ=1 the dummy state. A softcore-potential for the Lennard-Jones interactions with α_{LJ}=0.5 is used to prevent numerical instabilities and the simulations are 1 ns in length at each λ-state. Integration over $\langle dH/d\lambda \rangle_\lambda$ using the trapezoidal rule results in $-\Delta G_{D->N}$.

In addition to the corrections for electrostatic interactions, the standard state correction, ΔG_{std}, has to be applied in order to compare to experimental values. This can be done by

$$\Delta G_{std} = RT \ln\left(\frac{RTb^\circ \rho_S}{P^\circ}\right) \qquad \textbf{17}$$

where R is the ideal gas constant ($8.314 \cdot 10^{-3}$ kJ/mol/K) , T is the temperature (298K), b° is the molality (=1mol/kg), ρ_S is the density of the solvent at T (997 kg/m^3 for water and 788 kg/m^3 for methanol) and P° is the pressure (100 kJ/m^3). This results in standard state corrections of 7.36 and 7.95 kJ/mol for methanol and water simulations, respectively.

Once $\Delta G_{D->Q}$ has been calculated for all amino acid side chains and nucleobases in both water and methanol, the values of $\Delta G_{W->M}$(unb) can be determined with:

$$\Delta G_{W->M}(unb) = \Delta G_{D->Q}(M) - \Delta G_{D->Q}(W) \quad (18)$$

and are given in figure S3A and S3B for the amino acid side chains and nucleobases, respectively. The free energy difference between a nucleobase and a sidechain analog in the unbound state in water to the bound state in water is then:

$$\Delta G_{W,unb->M,bound} = \Delta G^{base}_{W->M}(unb) + \Delta G^{res}_{W->M}(unb) + \Delta G_{bind}(M) \quad (19)$$

where the values of ΔG_{bind}(M) can be found in figure 2B and the final results $\Delta G_{W,unb->M,bound}$ are shown in figure S4C.

Methods

Molecular dynamics (MD) simulations

The simulation box lengths used for MD simulations ranged between 3.7 and 4.1 nm and contained anywhere between 1600 and 2300 water molecules. The systems were equilibrated by applying position restraints to the solute (initial force constant of 2.5x10^4 kJ mol^{-1}) and generating velocities from the Maxwell-Boltzmann distribution at the temperature of 50 K. After 20 ps of simulation time, the force constant of the position restraints was lowered by a factor 10, whereas the temperature was increased by 50 K. This procedure was continued until a temperature of 250 K was reached. The last equilibration step was performed for 40 ps at the final temperature of 298 K, whereby position restraints were switched off, and instead the center-of-mass translation was removed every 1000 steps. The systems were weakly coupled *(4)* to an external bath with a temperature of 298 K and a coupling time of 0.1 ps, whereby the solute and solvent degrees of freedom were coupled to the heat bath independently. The pressure was kept constant at 1 atm using isotropic weak coupling with a compressibility of 7.51 x 10^{-4} (kJ mol^{-1} nm^{-3})$^{-1}$ and a relaxation time of 0.5 ps *(4, 5)*. Non-bonded interactions were calculated using a triple range cutoff scheme. Within the short-ranged cutoff of 0.8 nm, all interactions were calculated based on a pairlist which was generated every 5 steps. The interactions between 0.8 and 1.4 nm were calculated with every pairlist update and were kept constant between updates. Interactions beyond 1.4 nm were accounted for by a reaction field contribution with a dielectric permittivity of 61, which is appropriate for SPC water *(6)*. All simulations described above were repeated with methanol as solvent, whereby the cubic boxes were slightly larger (box lengths ranging between 4.1 and 4.9 nm) and filled with anywhere between 1300 and 1800 methanol molecules. The pressure was again kept constant at 1 atm using isotropic weak coupling, but now with a compressibility of 2.08 x 10^{-3} (kJ mol^{-1} nm^{-3})$^{-1}$ *(7, 8)*. The dielectric permittivity used to calculate the reaction field contribution for interactions beyond 1.4 nm was equal to 18.6 as is appropriate for the methanol model used in these simulations *(9)*.

Umbrella sampling

The restraining distances in US ranged between 0.2 and 1.9 nm, with a separation between individual steps of 0.1 nm. The weighted histogram analysis method (WHAM) (10) was used to unbias US simulations and to obtain PMF curves. In order to verify that convergence had been reached, the production runs were divided into 5 ns-long segments and the PMF was determined for each of them. In cases where the binding free energies calculated from these PMFs showed a discrepancy of more than 1.5 kJ mol^{-1}, the simulations at all windows were

prolonged by 5 ns, until convergence had been reached. Following this routine, the pairs GUA-HISB, THY-GLU and URA-GLU were prolonged up to 20, 20 and 15 ns, respectively, for the simulations in water. In methanol, the ADE-ARG, CYT-LYSH, GUA-ASN, GUA-GLU and GUA-HISB pairs were prolonged to a total simulation time of 15 ns or, in the case of CYT-ASP, 20 ns.

Matching between mRNA composition and cognate proteins' nucleobase affinity

The possibility of direct complementary interactions between mRNA and their cognate proteins was investigated based on the human proteome as extracted from the UniProtKB database (April 2013 release). Entries were required to be Swiss-Prot reviewed and have a protein existence annotation of at least "predicted" (*i.e.* proteins annotated as "uncertain" were excluded). The cross-references section of the UniProtKB entry provided the ENA (European Nucleotide Archive Database, http://www.ebi.ac.uk/ena) accession numbers of the mRNA sequences, from which the first one complying with the length criterion (RNA length = 3x protein length +3) was chosen. This procedure resulted in 16954 protein sequences together with their cognate mRNAs.

A binding preference scale was defined for each of the four RNA nucleobases (A, C, G and U) in both environments (water and methanol) based on the binding free energies between amino-acid side-chain analogs and nucleobases. For histidine, the average of the binding free energies for the two neutral forms (HISA/HISB) was chosen. Throughout the text, the scales are designated as, for example, G_{scl}(wat), which refers to the scale of binding free energies of amino-acid side-chain analogs to GUA in water.

The Pearson correlation coefficients between the sequence profiles capturing nucleobase content of mRNAs and nucleobase-affinity profiles of their cognate protein sequences were evaluated after applying a sliding-window averaging procedure to each sequence, where the window consisted of 21 side chains or codons. For this analysis, the minimum length of proteins was set to 43 side chains, corresponding to 2*w*+1 where *w* is the size of the averaging window. In cases where glycine or proline side chains were present along the protein sequence, these side chains were excluded from the average over the window in question, since the binding free energies could not be determined for these side chains using our current setup. The corresponding codons in the cognate mRNA were consequently also excluded from the window average. These window averages were thus calculated over fewer than 21 side chains/codons.

The significance of Pearson correlation coefficients was determined by random shuffling of the affinity scales. Each scale was shuffled 10^4 times and the Pearson correlations coefficients (R) between the nucleobase content of the mRNA and the nucleobase affinity of their cognate proteins were calculated for each of these shuffled scales. The P-values were then determined from the fraction of randomized scales which absolute value of R is greater than the R obtained with the original scale ($|R| > |R_{original}|$).

The significance of the effective free energies of interactions was evaluated by generating 10^6 randomized variants of each native mRNA sequence by exhaustively shuffling the positions of its codons. For each protein, the interaction energy was then not only determined for its cognate, native mRNA but also the shuffled ones. P-values were defined as the fraction of its randomized mRNAs that exhibited lower interaction free energies than the cognate mRNA.

REFERENCES

1. Kastenholz, M.A. and Hünenberger, P.H. (2006) Computation of methodology-independent ionic solvation free energies from molecular simulations. II. The hydration free energy of the sodium cation. *J. Chem. Phys.*, **124**, 224501.

2. Reif, M.M. and Hünenberger, P.H. (2011) Computation of methodology-independent single-ion solvation properties from molecular simulations. III. Correction terms for the solvation free energies, enthalpies, entropies, heat capacities, volumes, compressibilities, and expansivities of solvated ions. *J. Chem. Phys.*, **134**, 144103.

3. Reif, M.M., Hünenberger, P.H. and Oostenbrink, C. (2012) New Interaction Parameters for Charged Amino Acid Side Chains in the GROMOS Force Field. *J. Chem. Theory Comput.*, **8**, 3705–3723.

4. Berendsen, H.J.C., Postma, J.P.M., van Gunsteren, W.F., DiNola, A. and Haak, J.R. (1984) Molecular dynamics with coupling to an external bath. *J. Chem. Phys.*, **81**, 3684.

5. Haynes, W.M. ed. (2013) CRC Handbook of Chemistry and Physics 94th ed.

6. Heinz, T.N., van Gunsteren, W.F. and Hünenberger, P.H. (2001) Comparison of four methods to compute the dielectric permittivity of liquids from molecular dynamics simulations. *J. Chem. Phys.*, **115**, 1125.

7. Marcus, Y. (1998) The properties of solvents Hogg, P.G.T. (ed) John Wiley & Sons, Ltd, Chisester.

8. Caleman, C., van Maaren, P.J., Hong, M., Hub, J.S., Costa, L.T. and van der Spoel, D. (2012) Force Field Benchmark of Organic Liquids: Density, Enthalpy of Vaporization, Heat Capacities, Surface Tension, Isothermal Compressibility, Volumetric Expansion Coefficient, and Dielectric Constant. *J. Chem. Theory Comput.*, **8**, 61–74.

9. Walser, R., Mark, A.E., van Gunsteren, W.F., Lauterbach, M. and Wipff, G. (2000) The effect of force-field parameters on properties of liquids: Parametrization of a simple three-site model for methanol. *J. Chem. Phys.*, **112**, 10450–10459.

10. Kumar, S., Bouzida, D., Swendsen, R.H., Kollman, P.A. and Rosenberg, J.M. (1992) The weighted histogram analysis method for free-energy calculations on biomolecules. I. The method. *J. Comput. Chem.*, **13**, 1011–1021.
